# Gender Equality and Gender Inequalities in Self-Reported Health: A Longitudinal Study of 27 European Countries 2004 to 2016

**DOI:** 10.1177/0020731420960344

**Published:** 2020-10-05

**Authors:** Luis Roxo, Clare Bambra, Julian Perelman

**Affiliations:** 1 NOVA National School of Public Health, Universidade NOVA de Lisboa, Lisbon, Portugal; 2 Faculty of Medical Sciences, Institute for Population Health Sciences, Newcastle University, Newcastle upon Tyne, UK; 3 Public Health Research Centre, NOVA National School of Public Health, Universidade NOVA de Lisboa, Lisboa, Portugal

**Keywords:** Europe, health inequalities, gender, socioeconomic factors

## Abstract

Significant gender-based health inequalities have been observed across Europe, with women reporting worse health than men. Still, there has been little examination of how the gender–health gap has changed over time, and how it has been shaped by societal gender equality. We used data from the Statistics on Income and Living Conditions Eurostat database (EU-SILC), involving 2,931,081 participants aged 25–64, for 27 European countries. Logistic regressions were performed to model the association between self-reported bad health and gender, in general and over time. Analyses were stratified by employment, education, and clusters of countries according to levels of Gender Equality Index (GEI). Adjusting for age, year, and country, bad health was 17% more likely among women, but this disadvantage ceased after accounting for education and employment. Gender–health inequalities were larger among countries with higher GEI scores and among low-educated groups. The gender–health gap did not reduce significantly between 2004 and 2016, in general and within subgroups. Although societies are becoming more equal, persistent inequalities in employment and income still lead to sustained health differences between men and women.

Gender accounts for significant differences in health outcomes, albeit paradoxically. Although women live longer, this advantage does not translate into healthier years,^1,2^ as they steadily report worse health status and suffer from a higher burden of non-fatal and debilitating conditions.^3^

Aside from biological characteristics, several factors underpin these differences. Men and women are differently influenced by the social determinants of health, with women particularly hit by unfavorable socioeconomic and psychosocial factors.^4^ Some authors have described women’s morbidity disadvantage as a consequence of the patriarchy, which restrains women’s access to social and employment-related privileges and economic resources.^5–7^

Several studies have focused on gender-based inequalities in health at the country level, as gender gaps vary cross-nationally, likely as a result of country-specific conditions.^8^ Still, little is known about how gender differences in Europe have evolved in recent years and which country-specific factors have been driving these differences.

According to previous literature, there are contradictory hypotheses. Gender inequality is decreasing in European societies in its various dimensions. Policies to promote gender equality through several spheres of society have been implemented^6,9–11^ and have been connected to decreased health inequalities.^12^ Gender differences in educational attainment have ended, with a current advantage for women in secondary and tertiary education.^13^

Notwithstanding, equality has not been achieved yet. Women are less employed than men and more often work part-time.^14^ Segregation persists, with some jobs considered exclusive for women, mirroring the traditional gendered division of work.^15^ Women are employed in lower-paid sectors and receive lower salaries.^14^Time-use is unfairly distributed, and women are more often faced with the double burden of paid job and household/caregiving tasks.^14^ These persistent inequalities may also harm women’s health, through psychosocial mechanisms, by disappointing their expectations of an equal society.^16^

Additionally, there is evidence showing that the Great Recession – and austerity – might have widened health inequalities.^17,18^ It has been shown that women were more affected than men in countries that experienced a severe recession, especially in those that implemented austerity, such as Greece, Portugal, Spain, and the United Kingdom.^19–21^

Finally, increased societal gender equality may promote the adoption of unhealthy, masculine health-related practices.^8^ Gender gaps on smoking and alcohol consumption have been closing,^22,23^ and the prevalence and mortality of diseases such as lung cancer and cardiovascular disease have been rising among women, thus increasing gender-based inequalities in morbidity.^10,24^

Studies have tried to understand the links between societal gender equality and the gender gap in health, but results have depended on the outcomes, the measure of equality, and the period under analysis.^25^ Previous research has largely been cross-sectional, with little examination of the interaction between gender-based health inequalities and socioeconomic status.^5,26^ In this article, we provide the first analysis of the evolution of gender-based inequalities in self-reported health for 27 European countries from 2004–2016 and examine any association with changes in societal gender equality. We also examine how the evolution of gender-based inequalities varies by socioeconomic status.

## Methods

### Data Sources

Repeated cross-sectional data from the Statistics on Income and Living Conditions Eurostat (EU-SILC) survey were used. This instrument collects annual micro-data on income, poverty, labor, education, and health, using representative samples of European countries.^27^ Data from EU-SILC has previously been used in comparative research about health inequalities.^17,19^

We used individual data spanning 13 years, from 2004 to 2016, for 27 countries (Austria, Belgium, Bulgaria, Cyprus, Czechia, Denmark, Estonia, Finland, France, Germany, Greece, Hungary, Ireland, Italy, Latvia, Lithuania, Luxembourg, Malta, the Netherlands, Poland, Portugal, Romania, Slovakia, Slovenia, Spain, Sweden, and the United Kingdom). We excluded data from Iceland, Norway, Serbia, and Switzerland, because the gender index was not available for non-E.U. countries, and from Croatia, as data was only available for a small subset of years.

We excluded subjects above age 64 (n = 1,246,131), to consider the employment status of the working-age population; those under age 25 (n = 706,279), as they may not have finished their education^17^; and other participants with inconsistent age-related information (n = 85). We excluded observations with missing information on self-reported health (n = 493,137) and other variables used in our models (n = 15,148). Finally, we excluded those who reported being students (n = 37,599), disabled (n = 128,158), or military (n = 288), as their health assessment was not representative of the general population.

Our final sample included 2,931,081 participants. To adjust for non-response, we used the personal weights provided with the database.

### Dependent Variable

Self-reported health was obtained through the question “How is your health in general?” This variable has been shown to be associated with both physical and mental health problems^28^ and has been used in previous studies about gender–health inequalities.^29^ We recoded it as a binary variable and modeled bad health (original options “Bad” and “Very bad,” as opposed to “Very good,” “Good” and “Fair”).

### Explanatory Variables

Gender was measured as male or female sex. Societal gender inequality was assessed by the Gender Equality Index (GEI),^30^ an index aiming to monitor the evolution of gender equality across E.U. countries. The core index is formed by 6 domains (Work, Money, Knowledge, Time, Power, and Health), varying between 1 (total inequality) and 100 (full equality). We used data from 2005 and 2015 to understand the evolution of gender equality over time. The Health domain was excluded to avoid correlation with our dependent variable, so we calculated an arithmetic mean of the other 5 domains.

### Covariates

Age was used as a continuous variable for adjustments and categorically for characterization of the sample.

Socioeconomic status was measured by educational level and employment status. Educational level was coded as “Up to lower secondary education,” “Upper secondary education” (including post-secondary non-tertiary education), and “Tertiary education.” Employment status was coded as “Employed” (full-time or part-time), “Unemployed,” “Retired,” and “Out of labor” (those executing domestic tasks and other inactive persons).

Year of the survey and country were included as fixed effects. We added country-dummy variables to account for time-invariant, country-specific characteristics, such as cultural patterns,^31^ and year-dummy variables to estimate differences in our dependent variable over time.

### Data Analysis

We performed logistic regression models to model bad health as function of gender, first adjusting for age, year, and country, and then also for educational level and employment status.

To test if gender differences have changed over time, we then added the *gender × year* interaction term and calculated the yearly odds ratio (OR) for women (versus men). To understand the role of educational level and employment status on shaping the evolution of gender differences, analyses were then stratified by these 2 variables.

Afterward, we used K-means clustering analysis to classify countries by their societal gender equality, using the GEI score of 2005 and the difference between 2015 and 2005. We opted for a 5-cluster solution, as this was the first in which significant differences (*P* < .05) were noted for both variables, and named the clusters according to the GEI scores of 2005 and 2015.

To assess gender-based inequalities for each cluster, models were performed with the *gender × cluster* interaction. Finally, logistic regression models with *gender × year* interaction were performed with data stratified by cluster, to assess the evolution of gender inequalities for each cluster.

Data analysis was performed with STATA-13 and SPSS. Results were statistically significant when *P* < .05. When appropriate, 95% confidence intervals (95%CI) are presented. For the interaction terms, we calculated the OR and 95%CI by sequentially changing the reference categories of the model.^32^

### Ethics

Data collection respected the Helsinki Convention.^33^ All the analyses were performed with anonymized data, with no access to personal information.

## Results

### Description of the Sample

Women were slightly older than men, with a higher proportion among the 55–64 group (23.3%) and a lower representation among the 25–34 age group (23.2%) (Table 1). There were more women with up to lower secondary (26.2%) and tertiary education (28.2%) than men. There were more employed men (81.6%) than women (66.4%), whereas 17.9% of women were out of labor, against 1.9% of men.

**Table 1.** Characterization of the sample (% Observations) (N = 2,931,081).

	Total sample	Men (48.9%)	Women (51.1%)
Age groups			
25–34	23.4	23.6	23.2
35–44	27.5	27.8	27.2
45–54	26.4	26.5	26.3
55–64	22.7	22.1	23.3
Age in years (mean ± SD)	44.1 ± 11.0	44.2 ± 11.0	44.5 ± 11.1
Educational level			
Up to lower secondary	25.1	24.7	26.2
Upper secondary	47.1	48.2	45.6
Tertiary	27.8	27.1	28.2
Employment status			
Employed	73.9	81.6	66.4
Unemployed	8.5	9.0	8.1
Retired	7.6	7.5	7.6
Out of labor	10.1	1.9	17.9

### Gender-Based Health Inequalities in Europe

Women were 17% (OR = 1.17, 95%CI = 1.15–1.19) more likely to report bad health than men, adjusting for age, year, and country. When education and employment were factored in, women became less likely to report bad health than men (OR = 0.97, 95%CI = 0.96–0.99). Supplemental Table 1 provides results stratified by country.

**Figure 1.**
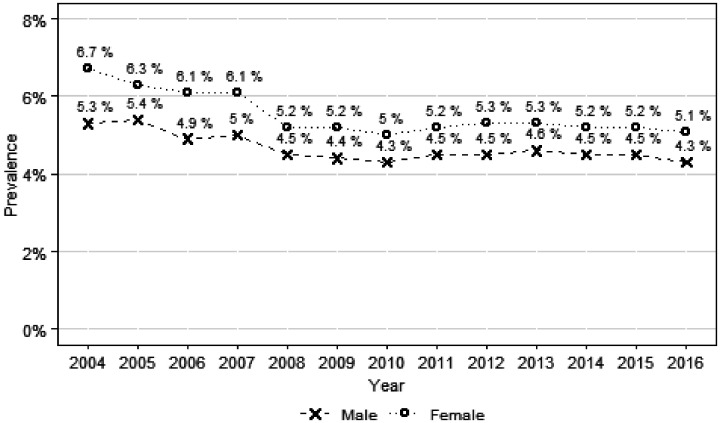
Prevalence of bad self-reported health, by gender, between 2004 and 2016.

The prevalence of bad health has decreased between 2004 to 2016 (Figure 1), among women (from 6.7% to 5.1%) and men (from 5.3% to 4.3%). The lowest prevalence was achieved in 2010 for both genders.

**Figure 2.**
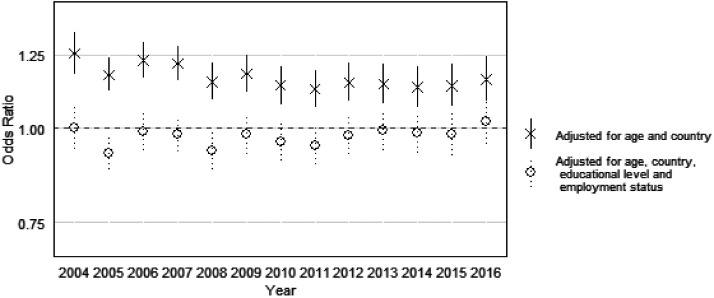
Risk of female bad self-reported health (OR, 95% CI), versus men (gender × year interaction), from 2004 to 2016.

Figure 2 shows the yearly women’s OR (versus men) obtained by the *gender × year* interactions. When adjusting for age, country, and year, women have higher odds for reporting bad health in every year. The OR for women’s bad health were larger in 2004 and smaller in 2010. However, all CI overlapped, so differences between years were non-significant. When adding education and employment to our models, women’s disadvantage ceased to exist in all the years, but again, with no significant differences between the years.

### Gender-Based Health Inequalities by Socioeconomic Status

**Figure 3.**
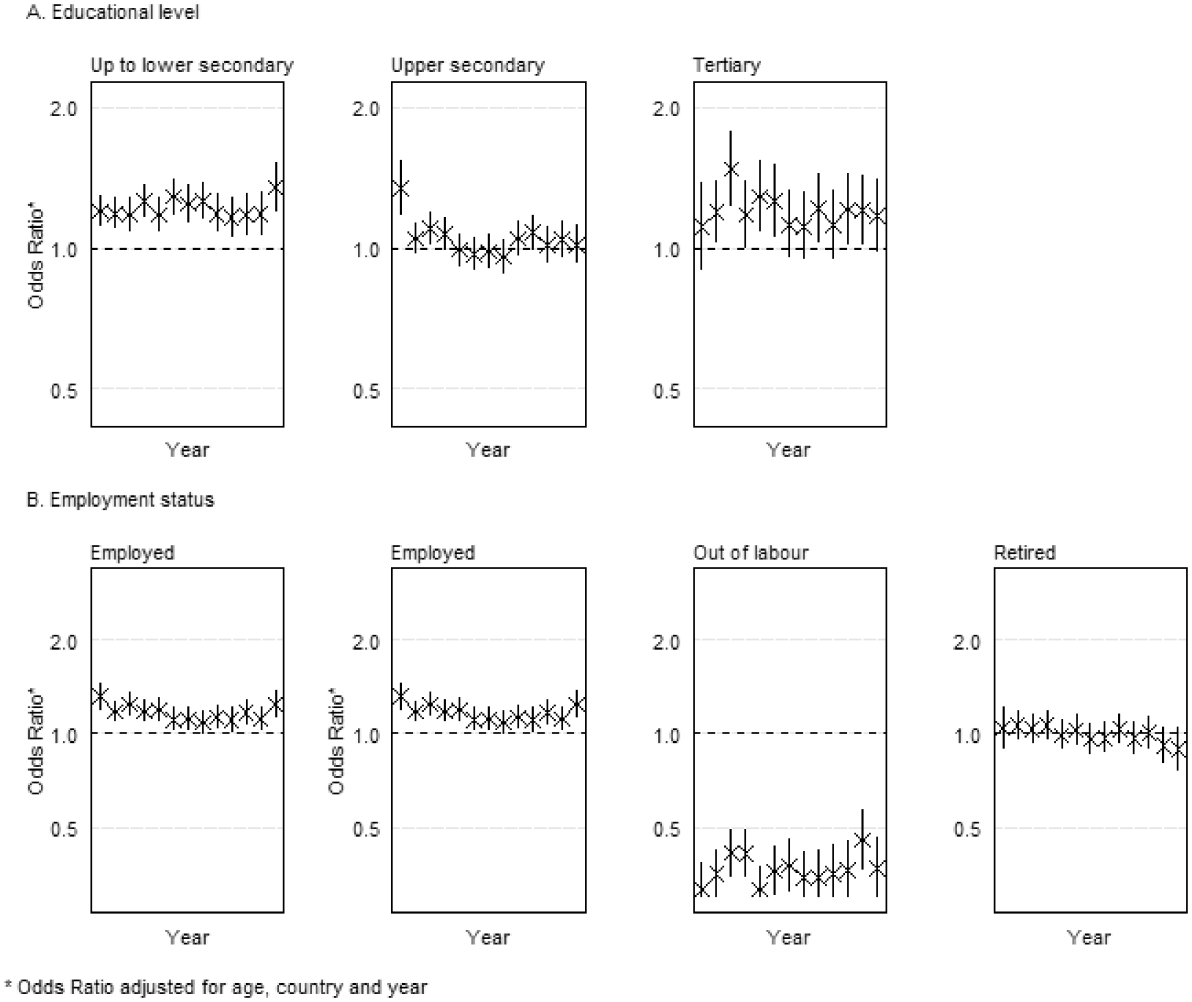
Risk of female bad self-reported health (OR, 95% CI), versus men (gender × year interaction), from 2004 to 2016, stratified by educational level and employment status.

Women’s health disadvantage was higher among low-educated groups, and this has not significantly decreased since 2004 (Figure 3A). Among this group, differences were larger in 2016 (OR = 1.34, 95%CI = 1.19–1.51). Among the upper secondary group, differences were only significant in 2004 (OR = 1.34, 95%CI = 1.17–1.53) and 2006 (OR = 1.10, 95%CI = 1.01–1.19). No significant gender differences were observed thereafter, and all confidence intervals overlapped after 2005. Among the group with tertiary education, OR were significant in several of the years under study, being larger in 2006 (OR = 1.48, 95%CI = 1.23–1.77). Still, no significant changes were found from 2004 to 2016.

Gender-based health inequalities among those employed have been stable (Figure 3B) and significant in every year but 2011. Among the unemployed, women had lower odds for reporting bad health between 2005 and 2008, with no significant differences since 2009. However, differences between years were not significant.

### Evolution of the Gender Equality Index

GEI’s mean score was 59.78 in 2005 and 63.44 in 2015. Greece scored worst in both years (49.2 and 51.38), whereas Sweden scored best (78.92 and 82.5). All countries had a higher GEI score in 2015, with the smallest increases observed in Hungary (0.32) and the United Kingdom (0.4), and the largest in Italy (7.94) and Cyprus (7.6).

K-means cluster analysis returned 5 clusters (Supplemental Figure 1). The cluster “Low-Low” (Bulgaria, Czechia, Estonia, Greece, Hungary, Lithuania, Poland, Romania, and Slovakia) had low equality in 2005 (52.93) and a small increase (2.12) until 2015. Cyprus, Italy, Latvia, Malta, and Portugal formed the cluster “Low-Medium.” This cluster had the lowest mean value in 2005 (52.87) and the largest increase to 2015 (6.02). The cluster “Medium-Medium” was formed by Austria, Germany, France, Ireland, Slovenia and Spain. This group’s mean GEI evolved from 62.42 to 67.71. Belgium, Finland, Luxembourg, Netherlands, and the United Kingdom composed the cluster “High-High,” with mean GEI of 69.04 in 2005 and growth of 2.42. The cluster “Very high-Very high” (Denmark and Sweden) had the highest equality (mean of 76.73 in 2005 and 79.65 in 2015).

Table 2 shows the results of the interaction *gender × cluster*. Women were more likely to report bad health in all clusters, adjusting for age, country, and year. Inequalities were larger in the cluster “Very high-Very high” (OR = 1.36, 95%CI = 1.26–1.48) and smaller in the cluster “Medium-Medium” (OR = 1.09, 95%CI = 1.06–1.12). After adjusting for employment and education, women’s disadvantage persisted in all clusters other than “Low-Low” and “Medium-Medium.” The largest inequalities remained in the cluster “Very high-Very high.”

**Table 2.** Distribution of bad self-reported health by gender (%) and risk of female bad self-reported health (OR and 95%CI), versus men (g*ender × cluster* interaction), by cluster of Gender Equality Index (2004–2016).

		Bad self-reported health(%)		Risk of female bad self-reported health (OR, 95% CI)	
Cluster	Countries	Men	Women	Adjusted for age^a^	Adjusted for age, educational level, and employment status^a^
Low–Low	Bulgaria, Czechia, Estonia, Greece, Hungary, Lithuania, Poland, Romania, Slovakia	5.5	6.7	1.18 (1.15–1.20)*	0.97 (0.95–1.00)*
Low–Medium	Cyprus, Italy, Latvia, Malta, Portugal	5.2	6.6	1.27 (1.23–1.31)	1.04 (1.01–1.08)*
Medium–Medium	Austria, Germany, Spain, France, Ireland, Slovenia	4.9	5.4	1.09 (1.06–1.12)*	0.91 (0.88–0.94)*
High–High	Belgium, Finland, Luxembourg, Netherlands, United Kingdom	2.7	3.4	1.28 (1.22–1.36)	1.07 (1.01–1.13)*
Very high–Very high	Denmark, Sweden	3.0	4.1	1.36 (1.26–1.48)	1.37 (1.26–1.48)

Abbreviations: CI, confidence interval; OR, odds ratio.

a All OR are also adjusted for country and year (fixed effects).

* *P* value < .05 (“Very high-Very high” as the reference category).

### Evolution of Gender Inequalities by Gender Equality Index Groups

Gender–health inequalities were significant in both 2004 and 2016 among clusters “Low-Low,” “Low-Medium,” and “High-High.” Among the cluster “Medium-Medium,” gender-based inequalities were only significant between 2004 and 2007. Regarding the cluster “Very high-Very high,” the gender–health gap started being significant in 2008, with the largest health inequalities in 2015. Overall, 95%CI overlapped in all clusters, so differences between years were not significant (Supplemental Figure 2).

## Discussion

This study aimed to understand the evolution of gender differences in self-reported health from 2004 to 2016 and to analyze how levels of societal gender equality might have shaped these changes. Women were more likely to report bad health, without any significant decrease of gender-based inequalities, in general and by sub-groups. Those in the least educated groups experienced the highest gender-related inequalities, whereas countries with greater societal gender equality did not experience a smaller health gap.

As expected, bad health was more common among women.^3,8,29^ Gender inequalities were fully explained by socioeconomic disparities between men and women, and, when educational level and employment were factored in, women even had a small health advantage compared to men. Previous research has stressed how gender differences in health measures are impacted by inequalities in the distribution of social determinants of health,^4,8,34^ especially the overrepresentation of women in groups with lower social resources.^4^

The prevalence of bad self-reported health has decreased for both genders, but no large gains were obtained, for men or women, since 2008. This is consistent with previous studies in Europe that show that the Great Recession terminated the positive trend in self-reported health,^35,36^ although some evidence has shown that the evolution depends on gender and age.^35,37^

Our main results show that gender inequalities in health persisted between 2004 and 2016, with a non-significant decrease. This happened regardless of the educational level, employment status, or cluster of societal gender equality. This may be related to the persistence of inequalities in society. Our data show that in 2016, women were still less likely to be employed than men, even though the proportion of women out of the labor force has been decreasing since 2004 (Supplemental Table 2). The employment gap has already been described as one of the main factors underlying gender inequalities in health and is associated with differences in outcomes such as chronic diseases and self-reported health.^3,4^ Female participation in the workforce may indeed have beneficial health effects for women, promoting their economic empowerment, social interaction, and self-esteem.^38,39^

Still, inequalities remain after women enter the workforce, as gender-based inequalities persist when solely employed people are included. This may be due to the enduring gender wage gap, regardless of women’s higher educational attainment,^40^ or the persistence of labor segregation, despite the increase of women in high-skilled, male-dominated occupations.^15^ Women are also burdened by combining their paid jobs with unequally distributed household and family-related activities.^14^

Gender-based health inequalities have persisted despite the increase of societal gender equality in all countries, as measured by the GEI. Gender equality typically involves 2 opposite movements toward non-traditional territories: women’s entrance into the job market and men’s increased participation in domestic and family-related tasks.^39^ Alone, the entrance of women in the job market may not be enough to end gender-based inequalities, as women may become more burdened by inequalities in the division of household labor.^25,39^

Our results also indicate larger gender differences among the group with lowest education, consistent with previous findings.^41^ Women with lower socioeconomic status may face specific threats to their health status. Gender gaps in employment rate and income are larger among low-educated persons.^42^ The domestic autonomy and bargaining power of these women may be limited by their lower individual income,^43^ increasing their vulnerability to intimate partner abuse.^44^ These women are also more likely to experience single parenthood and to raise children with less contact with the fathers.^45^ These women’s health disadvantage may create a vicious circle, in which poor health is influenced by low socioeconomic status and in turn contributes to it via downward social mobility or limited job opportunities/pay.^41,46^

Gender-based inequalities were significant for all the clusters of countries and larger in the cluster with higher societal gender equality. Previous research has shown that gender health inequalities persist in Scandinavian countries (particularly Sweden), despite the improvement in societal gender equality.^16^ This is the only cluster in which relative gender-based inequalities did not decrease after educational level and employment status were factored in, showing the limited explanatory relevance of these factors. This could be due to other structural factors such as the gender pay gap,^47^ labor market segregation,^15^ or women’s unfulfilled expectations of an equalitarian society.^16^ This paradox has some similarities with the Nordic public health puzzle, whereby despite having greater income equality, Scandinavian countries have larger relative health inequalities.^48^

### Strengths and Limitations

Our study provides information about the evolution of gender health inequalities by using a dataset of almost 3 million observations, with comparable longitudinal data from 27 countries.

To our knowledge, this is the first study using GEI to assess gender-based inequalities. Although some studies have used other measures of societal gender equality (Gender Inequality Index, Gender Empowerment Measure, or Gender Development Index),^25^ GEI provides a quantification of gender equality based on a much wider set of indicators. Although the index is not available for every year, we were able to capture the evolution of gender equality by using data from 2005 and 2015. Measures of gender equality are believed to differ among regions of the same country, particularly in decentralized states such as Germany and Spain.^49^ Still, no disaggregated data were available regarding the GEI or its components.

Self-reported health has been proven a reliable tool to assess health for both men and women,^28,34^ but it largely differs among countries and cultural settings.^8,26,50^ We believe that this bias may have been controlled by focusing on a measure of relative inequality (OR), instead of the prevalence of bad self-reported health.

## Conclusions

This study examined the evolution of gender inequalities in self-reported health in Europe between 2004 and 2016, in general and by socioeconomic status, and how levels of societal gender equality might have shaped any changes. Results show that women are more likely to report bad health, without any significant decrease of relative gender-based inequalities over time. Women in the least educated groups experience the highest gender–health gap. Differences in education and employment appear to be important in shaping gender-related inequalities in health, while countries with greater societal gender equality do not experience a smaller health gap. That is, our results do not support that higher levels of gender-equality inevitably lead to a smaller gender–health gap. Future research should examine why this is the case.

## Supplemental Material

sj-pdf-1-joh-10.1177_0020731420960344 - Supplemental material for Gender Equality and Gender Inequalities in Self-Reported Health: A Longitudinal Study of 27 European Countries 2004 to 2016 Click here for additional data file. Supplemental material, sj-pdf-1-joh-10.1177_0020731420960344 for Gender Equality and Gender Inequalities in Self-Reported Health: A Longitudinal Study of 27 European Countries 2004 to 2016 by Luis Roxo, Clare Bambra and Julian Perelman in International Journal of Health Services

sj-pdf-2-joh-10.1177_0020731420960344 - Supplemental material for Gender Equality and Gender Inequalities in Self-Reported Health: A Longitudinal Study of 27 European Countries 2004 to 2016 Click here for additional data file. Supplemental material, sj-pdf-2-joh-10.1177_0020731420960344 for Gender Equality and Gender Inequalities in Self-Reported Health: A Longitudinal Study of 27 European Countries 2004 to 2016 by Luis Roxo, Clare Bambra and Julian Perelman in International Journal of Health Services
