# Supplementary material for: Gender Equality and Gender Inequalities in Self-Reported Health: A Longitudinal Study of 27 European Countries 2004 to 2016
Source: Int J Health Serv. 2020 Oct 5;51(2):146–54. doi: 10.1177/0020731420960344 (PMC8114429; doi:10.1177/0020731420960344)
Supplement: sj-pdf-1-joh-10.1177_0020731420960344 - Supplemental material for Gender Equality and Gender Inequalities in Self-Reported Health: A Longitudinal Study of 27 European Countries 2004 to 2016 [file sj-pdf-1-joh-10.1177_0020731420960344.pdf]

Gender equality and gender inequalities in self-reported health: A longitudinal study of 27 European countries 2004 to 2016

Supplementary Table 1

Association of gender and self-reported health, for total sample and by country (2004 to 2016)

		Male	Female	Adjusted for age and year*		Adjusted for age, educational level, employment status and year*	
		%	%	OR (95%CI)		OR (95%CI)	
Total Sample				1.17 (1.15–1.19)	<0.001	0.97 (0.96–0.99)	0.003
Low-Low	Bulgaria	4.6	5.9	1.23 (1.14–1.33)	<0.001	1.09 (1.01–1.19)	0.036
	Czechia	5.2	5.9	1.12 (1.05–1.19)	0.001	0.89 (0.83–0.96)	0.002
	Estonia	5.6	6.0	0.96 (0.89–1.04)	0.327	1.03 (0.95–1.12)	0.451
	Greece	3.3	3.6	1.08 (0.99–1.17)	0.078	0.88 (0.79–0.97)	0.011
	Hungary	7.3	8.9	1.16 (1.11–1.22)	<0.001	1.03 (0.98–1.08)	0.282
	Lithuania	5.7	7.6	1.26 (1.16–1.38)	<0.001	1.29 (1.18–1.42)	<0.001
	Poland	6.5	7.6	1.11 (1.07–1.16)	<0.001	0.94 (0.90–0.98)	0.004
	Romania	4.2	5.8	1.32 (1.23–1.42)	<0.001	0.91 (0.84–0.98)	0.020
	Slovakia	6.3	8.2	1.16 (1.10–1.23)	<0.001	0.93 (0.88–0.98)	0.016
Low-Medium	Cyprus	3.8	4.0	1.09 (1.00–1.19)	0.053	0.79 (0.71–0.88)	<0.001
	Italy	4.4	5.3	1.20 (1.15–1.25)	<0.001	1.05 (1.00–1.10)	0.076
	Latvia	8.4	9.7	1.07 (1.01–1.13)	0.033	1.12 (1.05–1.20)	<0.001
	Malta	1.6	2.0	1.27 (1.10–1.46)	0.001	0.75 (0.58–0.98)	0.037
	Portugal	8.9	13.0	1.53 (1.46–1.61)	<0.001	1.36 (1.28–1.43)	<0.001
Medium-Medium	Austria	5.9	5.9	1.01 (0.94–1.07)	0.860	0.80 (0.74–0.84)	<0.001
	France	4.8	5.6	1.18 (1.12–1.25)	<0.001	1.02 (0.96–1.08)	0.426
	Germany	5.9	5.4	0.90 (0.86–0.95)	<0.001	0.72 (0.68–0.76)	<0.001
	Ireland	1.2	1.8	1.47 (1.23–1.74)	<0.001	1.20 (0.98–1.48)	0.079
	Slovenia	9.2	9.5	1.01 (0.95–1.07)	0.752	0.91 (0.86–0.97)	0.004
	Spain	3.4	5.1	1.46 (1.38–1.54)	<0.001	1.18 (1.10–1.26)	<0.001
High-High	Belgium	4.1	5.5	1.38 (1.29–1.49)	<0.001	1.06 (0.98–1.16)	0.149
	Finland	3.2	3.0	0.89 (0.81–0.98)	0.015	0.93 (0.84–1.02)	0.139
	Luxembourg	5.1	5.7	1.15 (1.05–1.26)	0.004	0.91 (0.81–1.02)	0.094
	Netherlands	2.1	3.0	1.47 (1.28–1.68)	<0.001	0.95 (0.81–1.11)	0.530
	UK	2.6	3.1	1.25 (1.15–1.35)	<0.001	1.08 (0.99–1.18)	0.068
Very high-Very high	Denmark	3.9	4.4	1.14 (1.02–1.27)	0.025	1.03 (0.91–1.16)	0.641
	Sweden	2.5	3.8	1.55 (1.39–1.73)	<0.001	1.59 (1.42–1.78)	<0.001

* Fixed-effects year

Supplementary Table 2

Evolution of education and employment, by gender (% observations, by year)

	2004	2005	2006	2007	2008	2009	2010	2011	2012	2013	2014	2015	2016
MEN													
Educational level													
Up to lower secondary	41.5	26.7	27.4	25.6	24.9	25.3	24.8	23.0	22.4	22.4	22.9	22.7	20.2
Upper secondary	37.7	46.0	47.9	50.5	49.7	48.8	48.8	49.8	49.6	49.5	48.1	47.3	47.2
Tertiary	20.8	27.3	24.8	23.9	25.4	25.8	26.4	27.2	28.0	28.2	29.0	30.0	32.6
Employment status													
Employed	79.8	81.7	83.0	82.8	83.3	81.4	81.0	81.1	81.0	79.9	80.7	81.8	83.0
Unemployed	8.2	7.9	7.3	6.7	6.7	8.5	9.2	9.6	10.2	11.4	11.1	10.3	9.1
Retired	9.5	8.5	7.8	8.8	8.1	8.0	7.9	7.6	6.9	6.8	6.3	6.1	6.3
Out of labor	2.5	2.0	1.9	1.8	1.9	2.1	1.9	1.8	1.8	1.8	1.9	1.8	1.8
WOMEN													
Educational level													
Up to lower secondary	43.2	28.7	29.7	28.4	27.4	27.3	26.1	24.4	23.4	23.1	23.7	23.0	20.6
Upper secondary	34.5	44.7	46.0	47.9	46.7	46.3	46.2	46.7	46.7	46.5	45.2	44.7	44.4
Tertiary	22.3	26.6	24.3	23.8	25.9	26.4	27.7	28.8	29.9	30.5	31.1	32.3	35.0
Employment status													
Employed	58.8	61.9	65.3	65.3	66.9	66.3	66.5	66.7	67.0	67.0	68.2	68.4	71.8
Unemployed	8.4	8.1	7.5	7.1	6.3	7.3	7.7	8.0	8.7	9.3	9.3	9.3	8.6
Retired	6.9	8.2	7.6	8.5	8.1	8.1	8.3	8.2	7.6	7.4	6.6	6.4	6.5
Out of labor	25.9	21.8	19.6	19.2	18.8	18.3	17.7	17.3	16.7	16.3	16.0	15.9	13.1
