# Supplementary material for: Gender Equality and Gender Inequalities in Self-Reported Health: A Longitudinal Study of 27 European Countries 2004 to 2016
Source: Int J Health Serv. 2020 Oct 5;51(2):146–54. doi: 10.1177/0020731420960344 (PMC8114429; doi:10.1177/0020731420960344)
Supplement: sj-pdf-2-joh-10.1177_0020731420960344 - Supplemental material for Gender Equality and Gender Inequalities in Self-Reported Health: A Longitudinal Study of 27 European Countries 2004 to 2016 [file sj-pdf-2-joh-10.1177_0020731420960344.pdf]

Supplementary Figure 1
Clusters of countries by Gender Equality Index (2005 and 2015)

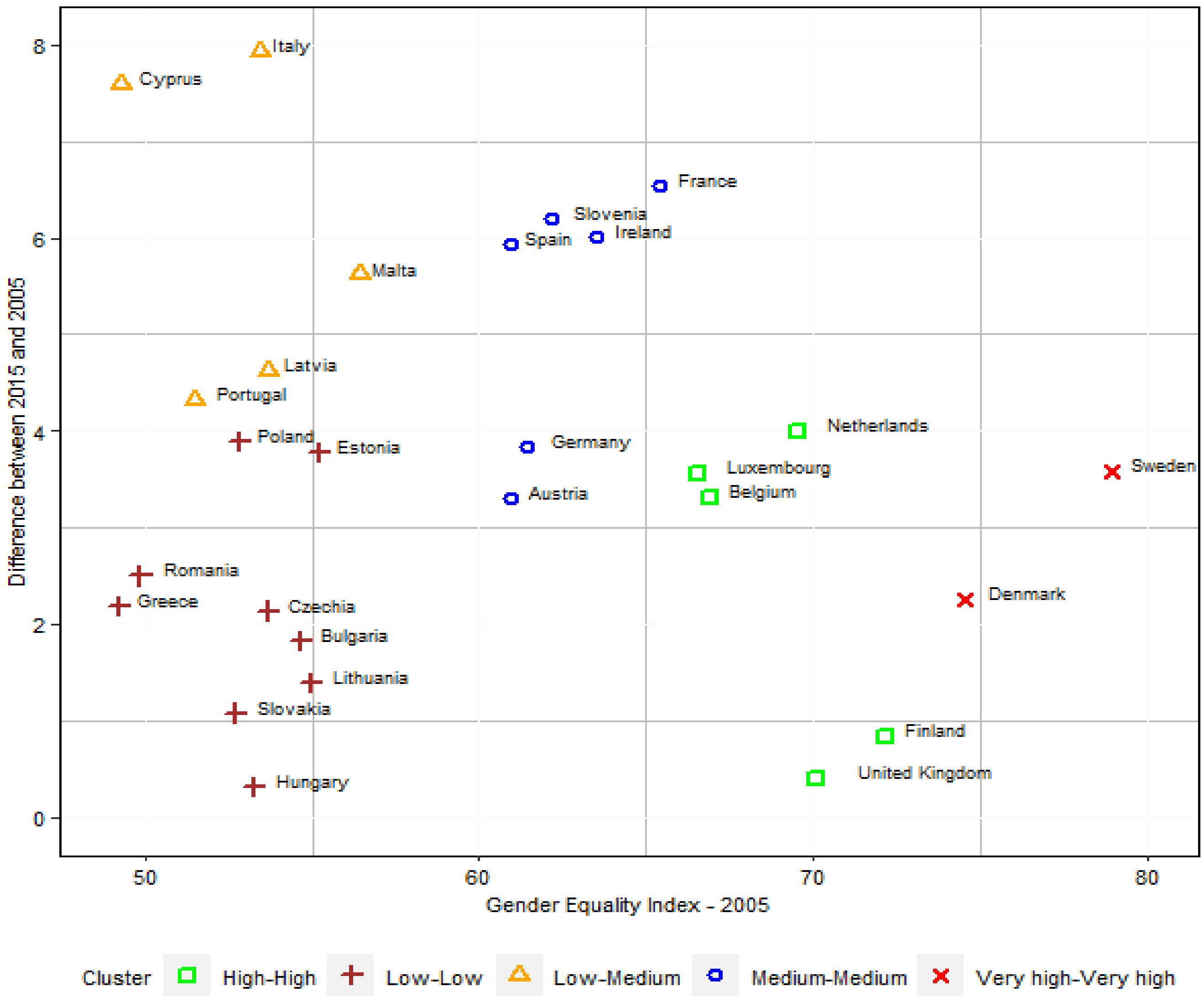

Supplementary Figure 2

Risk of female bad self-reported health (OR, 95%CI), versus men (gender*year interaction), from 2004 to 2016, stratified by clusters of Gender Equality Index

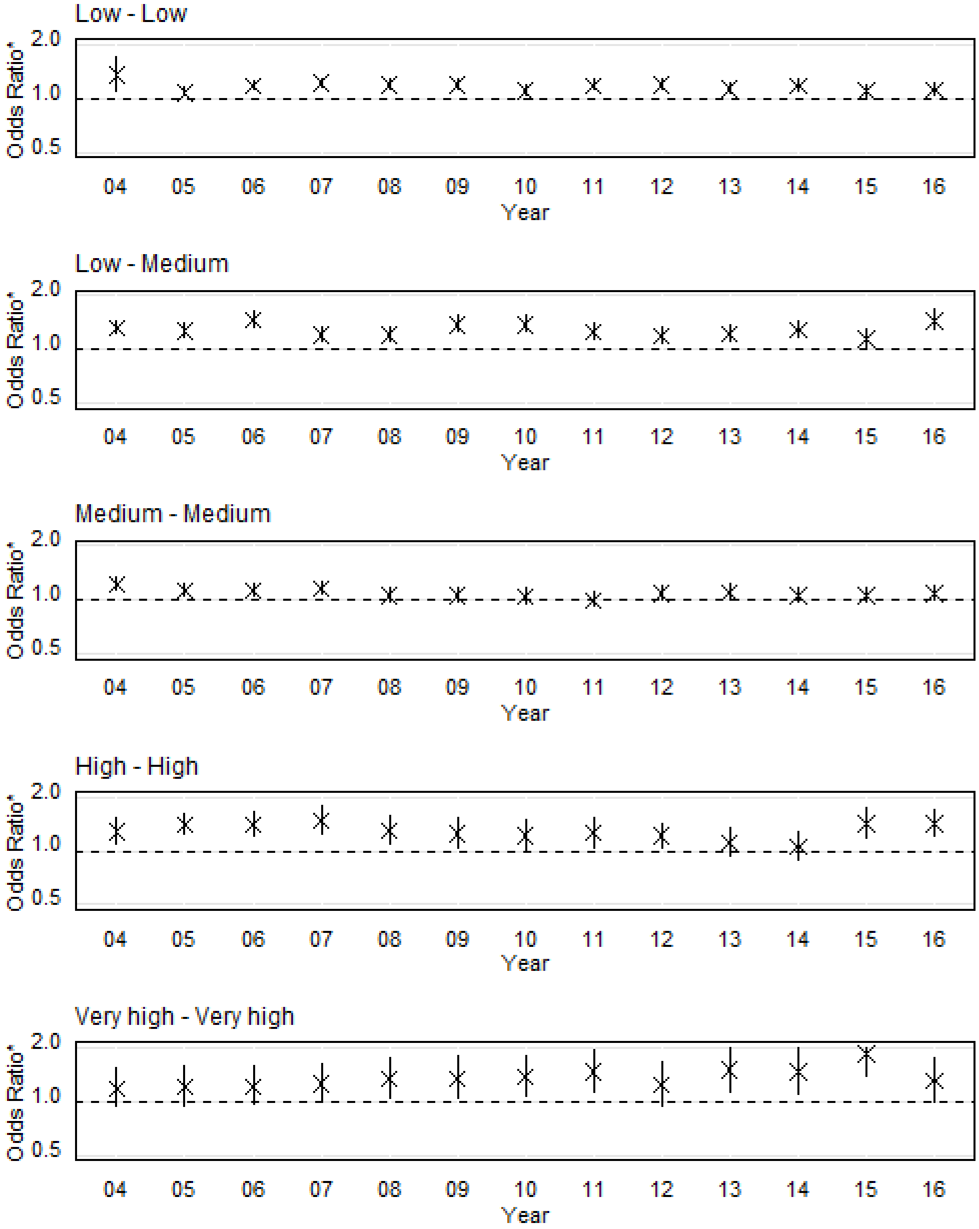

* Odds Ratio adjusted for age, country and year
